# Understanding *Haemophilus parasuis *infection in porcine spleen through a transcriptomics approach

**DOI:** 10.1186/1471-2164-10-64

**Published:** 2009-02-05

**Authors:** Hongbo Chen, Changchun Li, Mingdi Fang, Mengjin Zhu, Xinyun Li, Rui Zhou, Kui Li, Shuhong Zhao

**Affiliations:** 1 Key Lab of Agricultural Animal Genetics, Breeding, and Reproduction of Ministry of Education & Key Laboratory of Swine Genetics and Breeding of Ministry of Agriculture, Huazhong Agricultural University, Wuhan 430070, PR China; 2 Division of Animal Infectious Disease, State Key Laboratory of Agricultural Microbiology, Huazhong Agricultural University, Wuhan 430070, PR China; 3 Department of Gene and Cell Engineering, Institute of Animal Science, Chinese Academy of Agricultural Sciences, Beijing 100094, PR China

## Abstract

**Background:**

*Haemophilus parasuis *(HPS) is an important swine pathogen that causes Glässer's disease, which is characterized by fibrinous polyserositis, meningitis and arthritis. The molecular mechanisms that underlie the pathogenesis of the disease remain poorly understood, particularly the resistance of porcine immune system to HPS invasion. In this study, we investigated the global changes in gene expression in the spleen following HPS infection using the Affymetrix Porcine Genechip™.

**Results:**

A total of 931 differentially expressed (DE) transcripts were identified in the porcine spleen 7 days after HPS infection; of these, 92 unique genes showed differential expression patterns based on analysis using BLASTX and Gene Ontology. The DE genes involved in the immune response included genes for inflammasomes (*RETN*, *S100A8*, *S100A9*, *S100A12*), adhesion molecules (*CLDN3*, *CSPG2*, *CD44*, *LGALS8*), transcription factors (*ZBTB16*, *SLC39A14*, *CEBPD*, *CEBPB*), acute-phase proteins and complement (*SAA1*, *LTF*, *HP*, *C3*), differentiation genes for epithelial cells and keratinocytes (*TGM1*, *MS4A8B*, *CSTA*), and genes related to antigen processing and presentation (*HLA-B*, *HLA-DRB1*). Further immunostimulation analyses indicated that mRNA levels of *S100A8*, *S100A9*, and *S100A12 *in porcine PK-15 cells increased within 48 h and were sustained after administration of lipopolysaccharide (LPS) and Poly(I:C) respectively. In addition, mapping of DE genes to porcine health traits QTL regions showed that 70 genes were distributed in 7 different known porcine QTL regions. Finally, 10 DE genes were validated by quantitative PCR.

**Conclusion:**

Our findings demonstrate previously unrecognized changes in gene transcription that are associated with HPS infection *in vivo*, and many potential cascades identified in the study clearly merit further investigation. Our data provide new clues to the nature of the immune response in mammals, and we have identified candidate genes that are related to resistance to HPS.

## Background

The pig is an important agricultural animal and is an excellent mammalian model for biomedical research [1]. The infection caused by *Haemophilus parasuis *(HPS) has become an increasing threat in early-weaned pigs and in pig herds of high health status. HPS is a Gram-negative bacillus that causes Glässer's disease, which is characterized by fibrinous polyserositis, meningitis and arthritis [2]. Other clinical manifestations, e.g., acute pneumonia, acute fasciitis and myositis, acute septicemia and the formation of microthrombi resulting from disseminated intravascular coagulation, have also been reported [3-5]. The disease can also lead to severe sequelae, such as abortion in sows and chronic stringhalt in boars, in pigs that survive the acute infection. Cases of invasive disease caused by HPS have been reported in many countries, including China, Japan, the USA, Germany, Spain, and Australia [6].

Despite the devastating losses it causes to the pig industry, not much is known about the pathogenesis and the host genes that respond to HPS infection. Studies of several putative virulence factors of HPS, which have involved the reproduction of the disease in systems that mimic the *in vivo *environment, have increased our understanding of the the regulatory mechanisms [7-9]. However, a major obstacle to a better understanding of the pathogenesis has been the lack of information about the molecular mechanisms that mediate the interactions between HPS and the host under natural conditions *in vivo*. Mannan-binding lectin A (MBL-A) has been found to bind HPS, which suggests that it may have antibacterial functions [10]. Notably, a recent paper on the pathogenesis of meningitis induced by HPS has been published during the preparation of our manuscript [11]. This work demonstrated that HPS lipooligosaccharide plays a contributory role in the adhesion of HPS to porcine brain microvascular endothelial cells (PBMEC). The authors also found that the permeability of the blood-brain barrier may have been increased as a result of the induction of IL-6 and IL-8 in PBMEC following HPS infection. The above authors are pioneers in the study of HPS infection, but the characterization of the immune response involved in host protection is still an area in urgent need of investigation.

The presence of pseudorabies virus (PRV) infection may allow HPS to proliferate in the lungs [12], but the results of many other studies have indicated that PRV has no influence on the incidence of HPS infection. [13-16]. In Korea, mixed infection with porcine circovirus type 2 (PCV-2) and HPS was the most prevalent combination associated with postweaning multisystemic wasting syndrome [17]. We presume that the same porcine effector molecules might be involved in infections with different microorganisms. Inflammation is a typical manifestation of HPS invasion. Thus, an understanding of the differentially expressed (DE) genes that are involved in the inflammatory response may contribute to a better understanding of the pathogenesis of HPS infection. In this study, we investigated the gene expression patterns in the spleen followingHPS infection through a transcriptomics approach. The significant DE genes involved in the immune response were found to encode proteins related to inflammation, cell adhesion, regulation of transcription, differentiation of epithelial cells and keratinocytes, acute-phase response, and antigen processing and presentation. Our results present novel results regarding the transcriptional profile in pig spleen following HPS infection. These results will lead to therapies for HPS and candidate genes for HPS resistance as well as provide fundamental information regarding mammalian immune response mechanisms.

## Results

### Spleen transcriptome analyses

The transcriptome of non-infected porcine spleens was determined, and 13,857 probesets were identified to have expression in this tissue. The total number of transcripts expressed in infected spleens during HPS infection was also calculated. Expression was detected for 13,833 transcripts (58% of all probesets) in the spleen during infection. A total of 14,729 transcripts were expressed in infected and non-infected porcine spleens (see Additional file 1).

### Identification of DE genes in porcine spleens following HPS challenge

**Figure 1:**
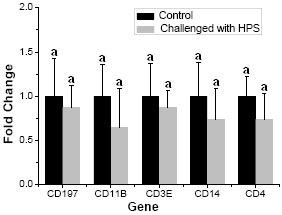
**QPCR evaluation on cell migrations in spleen following HPS infection**. The same letter "a" above the bars for each marker suggested a no significant fold change between the controls and the infected spleens. Fold change is calculated based on the mean intensity value from 3 donors within each group by using the comparative Ct method. Significant levels (0.05) were analyzed by T-test.

Significant migration of cell could result in masking of gene expression levels. The fact that cells, particularly immune cells, constantly migrate in and out of the spleen during HPS infection *in vivo *should be considered. The expression of five marker genes (Table 1) was evaluated by QPCR analyses, and no evidence was obtained that expression levels had changed as a result of significant migration of cells (Figure 1).

**Table 1:** Primers used for QPCR evaluation on cell migrations

Gene	Marker for cell types	Primer sequence (5'-3')	Target size (bp)	Tm (°C)^a^
*CD197*	T cell dendritic cell	Forward: TGTGGTTTCAGCAGCCAAGAG Reverse: GCCGATGTAGTCGTCCGTGA	130	59.8
*CD11B*	Macrophage Granulocyte	Forward: CAACCTGGGTCAGAGGAAGC Reverse: CAGACAGCGATGGAGCAGTT	207	62
*CD3E*	T cell	Forward: ACCTCTTAGTTCCTCCCTTTG Reverse: TGCCAGCATTTACCCAGTC	137	59.8
*CD14*	Macrophage Granulocyte	Forward: GCAGAGGCTTTGAGGACCTTATC Reverse: GCTGCGGATGCGTGAAGTT	154	62
*CD4*	T cell NK cell	Forward: TCTGCGAAGTGGAAGACAAG Reverse: GCTCTTGACGTCATTCTTGC	179	59.8
*RPL32*^b^		Forward: CGGAAGTTTCTGGTACACAATGTAA Reverse: TGGAAGAGACGTTGTGAGCAA	94	59.8–62

^a^The annealing temperature represents the optimal temperature during quantitative PCR. ^b^RNA levels of RPL32 was assayed for normalization during quantitative PCR.

**Figure 2:**
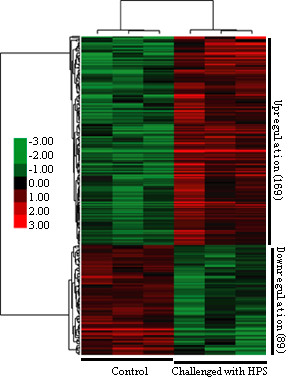
**A hierarchical cluster of 258 transcripts following HPS infection in 30-day-old piglet spleen**. Each row represents a separate transcript and each column represents a separate piglet. Color legend is on the left, red indicates increased transcript expression levels, whereas green indicates decreased levels compared with normal samples. Up-/down- regulated response transcripts are highlighted on the right with the No. in each cluster in parentheses. Fold change is calculated based on the mean intensity value from 3 donors within each group.

The global expression profile of porcine spleen 7 days after challenge with HPS was compared with that of the control group. After quantile normalization and statistical analyses, 931 transcripts were identified at the *p *< 0.05 level (see Additional file 2). Taking a fold change (FC) of two or greater and the *p *< 0.05 significance level as the criteria, a total of 258 transcripts showed differential expression. A clear expression pattern emerged after hierarchical clustering analyses of the 258 transcripts (*p *< 0.05, FC≥2). A set of 169 transcripts with similar responses to HPS infection belonged to the up-regulated group, and another set of 89 transcripts belonged to the down-regulated group (Figure 2).

**Figure 3:**
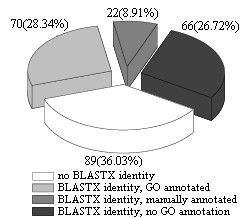
**Percentage distribution of unique genes from 258 differentially regulated transcripts after BLASTX searches and annotation**. 158 unique genes had significant similarities based on BLASTX searches. 92 (70+22) unique genes had been annotated by Biology Process (BP) Classification. Percentage of each part was marked in the parentheses.

**Figure 4:**
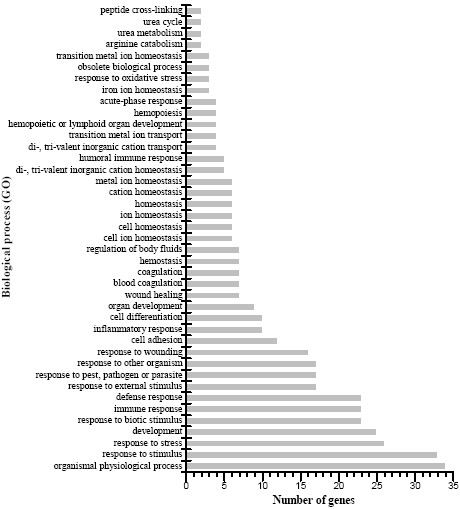
**Categories of 70 unique genes (FC≥2, *p *< 0.05) based on biological process GO term**. Many categories shared the same transcripts.

Of the 258 DE transcripts, putative identities could be determined for 166 transcripts, based on BLASTX searches. Annotation of the 166 transcripts was carried out using the Database for Annotation, Visualization and Integrated Discovery (DAVID) and by searching against the GenBank database. We found that a few probes on the microarray were derived from the same UniGene. Therefore, of the 166 DE pig transcripts, 158 are believed to represent unique genes. A total of 92 unique genes were annotated by Biology Process (BP) Classification (Figure 3); of these, 70 genes were grouped into 41 categories based on biological process Gene Ontology (GO) terms (Figure 4). Another 22 genes were annotated manually according to their potential BP by referring to recent publications. Analyses of GO and the results of pathway prediction also indicated that there were 8 categories (119 transcripts) identified by Cellular Component (CC) Classification, and 32 categories (131 transcripts) identified by Molecular Function (MF) Classification (data not shown). The majority of the genes related to the immune response could be assigned into numerous subcategories related to the inflammatory response, the acute-phase response, the humoral immune response, cell differentiation, cell adhesion, oxidative stress, homeostasis, transcription factors, and the response to wounding. Another important set of genes that are related to development was also identified (Table 2). Genes for which putative identities could be determined based on BLASTX searches, but that are not involved in our annotations, and genes with no BLASTX identity are listed in Additional file 3.

**Table 2:** The 92 annotated genes in piglet spleen following HPS infection^a^

Functional classification	Gene Discription^b^	GenBank ID	FC^c^	*p*-value^d^
**Inflammatory response**	*Resistin*	NM_020415	*25.9*	*2.70E-02*
	S100 calcium binding protein A9	NM_002965	21.8	1.60E-03
	S100 calcium binding protein A12	NM_005621	16.6	1.29E-02
	S100 calcium binding protein A8	NM_002964	8.4	2.29E-02
	*Chitinase 3-like 1*	NM_001276	*6.3*	*1.45E-02*
	*Calpain; small subunit 1*	NM_001003962	*5.7*	*2.50E-03*
	Interleukin 1 receptor accessory protein	NM_002182	4.6	6.00E-04
	Complement component 3	NM_000064	3.3	4.51E-02
	*Solute carrier family 2, member 3*	NM_006931	*3.3*	*4.05E-02*
	Interleukin 10 receptor; beta	NM_000628	2.8	3.47E-02
	*ADAM metallopeptidase with thrombospondin type 1 motif 1*	NM_006988	*2.6*	*2.24E-02*
	Histone deacetylase 9	NM_014707	0.5	7.00E-04
	Chemokine (C-C motif) ligand 5	NM_002985	0.4	3.30E-03
**Acute-phase response**	Serum amyloid A1	NM_000331	23.9	1.35E-02
	Lactotransferrin	NM_002343	13.0	2.26E-02
	Angiotensinogen	NM_000029	10.2	6.50E-03
	Haptoglobin	NM_005143	10.0	1.36E-02
	Ceruloplasmin/ferroxidase	NM_000096	4.9	4.09E-05
**Humoral immune response**	CD163 antigen	NM_203416	4.6	3.56E-02
	*Extracellular matrix protein 1*	NM_004425	*2.7*	*9.90E-03*
	Lysozyme	NM_000239	2.3	4.20E-03
	CD2 (cytoplasmic tail) binding protein 2	NM_006110	0.4	2.79E-02
**Cell differentiation**	Transglutaminase 1	NM_000359	22.3	1.62E-02
	*Membrane-spanning 4-domains; subfamily A; member 8B*	NM_031457	*20.5*	*1.61E-02*
	*Cystatin A*	NM_005213	*13.1*	*3.78E-02*
	Serpin peptidase inhibitor, clade E, member 2	NM_006216	3.6	2.74E-02
	Secreted frizzled-related protein 1	NM_003012	2.3	3.23E-02
	Growth arrest and DNA-damage-inducible; gamma	NM_006705	2.0	2.79E-02
	Carnitine deficiency-associated; expressed in ventricle 1	NM_014055	0.4	2.24E-02
	Myeloid leukemia factor 1	NM_022443	0.4	1.61E-02
	Stathmin-like 2	NM_007029	0.2	4.11E-02
	Mal; T-cell differentiation protein	NM_022438	0.2	2.82E-02
**Cell adhesion**	Claudin 3	NM_001306	9.7	2.34E-02
	*Chondroitin sulfate proteoglycan 2/versican*	NM_004385	*9.3*	*7.50E-03*
	*Lectin, galactoside-binding, soluble, 8*	NM_006499	*2.8*	*7.10E-03*
	CD44 antigen	NM_001001392	2.5	5.50E-03
	Immunoglobulin superfamily containing leucine-rich repeat	NM_201526	2.3	3.44E-02
	Discs; large homolog 5	NM_004747	2.2	2.05E-02
	Thrombospondin 1	NM_003246	2.2	3.49E-02
	Collagen; type XV; alpha 1	NM_001855	2.0	1.03E-02
	Nephronophthisis 1	NM_000272	0.5	2.18E-02
	Nuclear receptor subfamily 3; group C; member 2	NM_000901	0.4	3.18E-02
	Fibronectin leucine rich transmembrane protein 3	NM_198391	0.4	3.62E-02
	PDZ domain containing 2	NM_178140	0.3	8.10E-03
**Response to oxidative stress**	Superoxide dismutase 2; mitochondrial	NM_000636	3.8	6.10E-03
	Dual specificity phosphatase 1	NM_004417	2.1	2.05E-02
**Homeostasis**	Transcobalamin I	NM_001062	17.7	3.06E-02
	*Solute carrier organic anion transporter family, member 2A1*	NM_005630	*3.0*	*5.00E-04*
	*ATP-binding cassette, sub-family A (ABC1), member 5*	NM_018672	*2.6*	*2.48E-02*
**Transcription factor**	*Zinc finger and BTB domain-containing protein 16*	Q05516	*5.6*	*9.20E-03*
	*CCAAT/enhancer binding protein (C/EBP), delta*	NM_005195	*4.7*	*4.59E-02*
	*Solute carrier family 39 (zinc transporter), member 14*	NM_015359	*4.5*	*3.66E-02*
	CCAAT/enhancer binding protein (C/EBP), beta	NM_005194	3.1	3.00E-02
	Nuclear factor; interleukin 3 regulated	NM_005384	2.3	4.70E-03
**Response to wounding**	Macrophage stimulating 1	NM_020998	2.9	3.31E-02
	Coagulation factor III	NM_001993	2.8	1.74E-02
	Annexin A8	NM_001630	2.4	4.19E-02
	Coagulation factor XIII; A1 polypeptide	NM_000129	2.4	4.59E-02
**Other responses to stimulus**	GTP binding protein overexpressed in skeletal muscle	NM_181702	3.6	1.73E-02
	*Period homolog 1*	NM_002616	*2.6*	*7.50E-03*
	Interleukin 4 receptor	NM_000418	2.2	2.57E-02
	Major histocompatibility complex; class II; DR beta 1	NM_002124	0.5	4.18E-02
	Guanylate binding protein 1; interferon-inducible; 67 kDa	NM_002053	0.5	8.70E-03
	DnaJ	NM_012266	0.5	3.60E-03
	Pituitary tumor-transforming 1	NM_004219	0.5	5.00E-04
	Potassium voltage-gated channel; subfamily H	NM_172057	0.5	4.33E-02
	Major histocompatibility complex; class I; B	NM_005514	0.3	6.00E-04
**Development**	*Cofilin 1*	NM_005507	*19.4*	*3.18E-02*
	P8 protein	NM_012385	5.4	1.57E-02
	Neuron navigator 1	NM_020443	3.4	1.92E-02
	apolipoprotein D	NM_001647	2.6	8.00E-03
	*Protein tyrosine phosphatase; non-receptor type 1*	NM_002827	*2.3*	*4.58E-02*
	Putative insulin-like growth factor II associated prote	NM_001007139	2.2	2.60E-03
	FYVE; RhoGEF and PH domain containing 4	NM_139241	2.2	4.74E-02
	*Slingshot homolog 2*	NM_033389	*2.2*	*3.00E-04*
	3-phosphoadenosine 5-phosphosulfate synthase 2	NM_001015880	2.1	2.89E-02
	Epimorphin	NM_194356	0.5	4.16E-02
	Chondrolectin	NM_024944	0.5	2.66E-02
	Ecotropic viral integration site 5	NM_005665	0.5	1.52E-02
	SMAD; mothers against DPP homolog 5	NM_001001419	0.5	4.20E-02
	*Stearoyl-CoA desaturase*	NM_005063	*0.5*	*1.16E-02*
	*Ankyrin 3, node of Ranvier*	NM_001149	*0.5*	*9.40E-03*
	Insulin-like growth factor 2	NM_000612	0.4	1.76E-02
	SRY	NM_003108	0.4	4.06E-02
	Chromosome 11 open reading frame 8	NM_001584	0.4	3.25E-02
	Actin; alpha; cardiac muscle	NM_005159	0.3	8.10E-03
	Cellular retinoic acid binding protein 1	NM_004378	0.1	4.34E-02
	Erythroid associated factor	NM_016633	0.01	6.70E-03
**Urea cycle**	Arginase; type II	NM_001172	2.1	1.41E-02
	Arginase; type I	NM_000045	0.2	2.53E-02
**Organismal physiological process**	v-maf musculoaponeurotic fibrosarcoma oncogene homolog F	NM_152878	5.2	2.00E-04
	*Uridine phosphorylase 1*	NM_181597	*4.4*	*1.94E-02*

^a^Up/down-regulated genes which putative functions assigned based on GO term and manual annotation. Manual annotations were listed in italics. Many genes could be classified into multiple categories but only the most specific biology processes were listed. ^b^Top informative BLASTX hit. ^c^Fold change, gene expression level following infection compared to the control. "≥2" represents up regulation (infection/control), "≤0.5" represents down regulation. ^d ^Significance level of differential expression for a particular gene.

### Inflammasomes

Analysis of global mRNA expression in the porcine spleen using the Affymetrix microarray identified a large set of DE genes that are involved in the inflammatory response (Table 2). *RETN *(resistin), an adipocytokine, which was up-regulated 25.9-fold in our assay, has been proven recently to be an important mediator of the immune response and inflammation [18]. Another three members of the S100 family, *S100A8 *(S100 calcium binding protein A8, 8.4-fold), *S100A9 *(S100 calcium binding protein A9, 21.8-fold), and *S100A12 *(S100 calcium binding protein A12, 16.6-fold), were also remarkably up-regulated. Other genes whose expression levels were significantly higher (more than 3-fold increased) in the inflammatory response were *CHI3L1 *(chitinase 3-like 1), *CAPNS1 *(calpain small subunit 1), *IL1RAP *(interleukin 1 receptor accessory protein), *C3 *(complement component 3), and *SLC2A3 *(solute carrier family 2 member 3), which were increased by 6.3-, 5.7-, 4.6-, 3.3-, and 3.3-fold, respectively. Additionally, *IL-10Rβ *(cell surface interleukin 10 receptor beta), which is known to be involved in binding IL-10 showed higher expression levels in the HPS-infected spleens (2.8-fold). In contrast, *HDAC9 *(histone deacetylase 9) and *CCL5 *(chemokine ligand 5) were down-regulated, at 2-fold and 2.5-fold respectively, following HPS infection.

### Acute-phase proteins and induction of genes associated with the humoral immune response

The expression of genes known to participate in the acute-phase response and the humoral immune response (Table 2) included the most highly up-regulated gene, *SAA1 *(serum amyloid A1) which is also associated with inflammation, at 23.9-fold. *LTF *(the iron-binding protein lactotransferrin, 13.0-fold) and the macrophage scavenger receptor *HP *(haptoglobin, 10.0-fold) showed increased expression levels in infected porcine spleen. Together with that of *HP*, elevation of *CD163 *(CD163 antigen, 4.6-fold) suggests that intracellular signalling leading to increased hemoxygenease activity and the release of anti-inflammatory cytokines may have occurred after infection. Interestingly, bacteriocidal *LYZ *(lysozyme), which is known to be synergistic with *LTF*, was also up-regulated (2.3-fold), indicating activation of the antimicrobial capability of the host. Other highly induced genes included *AGT *(the vascular inflammasome angiotensinogen, 10.2-fold) and another *CP *(ceruloplasmin) that is related to iron homeostasis, also named ferroxidase, which was 4.9-fold higher compared with control levels.

### Genes for cell differentiation and cell adhesion

The gene set related to cell differentiation included 10 DE genes (Table 2), of which the skin barrier gene *TGM1 *(transglutaminase 1) showed the highest mRNA expression levels (22.3-fold) after infection. The new MS4A family member, *MS4A8B *(membrane-spanning 4-domains subfamily A member 8B), was found at levels 20.5-fold higher than in normal controls. Additionally, another gene that was annotated manually, *CSTA *(cystatin A), has recently been demonstrated to play an important role in cells of the immune system. It was up-regulated 13.1-fold in our assay after infection. Of note, the candidate linker protein that is involved in T-cell signal transduction, *MAL *(T-cell differentiation protein), and a newly identified gene, *STMN2 *(stathmin-like 2), showed dramatic down-regulation following HPS infection (5-fold). Cell adhesion molecules (CAMs) have been implicated in the regulation of a wide variety of fundamental cellular processes, not only cell adhesion, but also cell polarization, survival, movement, and proliferation [19]. Our results revealed a relatively small number of CAMs following HPS infection. *CLDN3 *(claudin 3) and *CSPG2 *(chondroitin sulfate proteoglycan 2/versican) were the two genes with the highest FCs (9.7- and 9.3-fold respectively). *LGALS8 *(lectin, galactoside-binding, soluble, 8) is a CAM that is related to apoptosis, and it was found to be increased 2.8-fold in our assay. Another important cell-surface glycoprotein *CD44 *(CD44 antigen) was found to be 2.5-fold increased after HPS infection.

**Table 3:** Primers used for QPCR validation and additional expression profiling

Gene	Primer sequence (5'-3')	Target size (bp)	Tm (°C)^a^
*RETN*	Forward: CCTCTTCCTCCCAACCCTG Reverse: GAGGTGACACTCCGGCATT	150	62
*S100A12*	Forward: GGCATTATGACACCCTTATC Reverse: GTCACCAGGACCACGAAT	168	60
*S100A9*	Forward: CCAGGATGTGGTTTATGGCTTTC Reverse: CGGACCAAATGTCGCAGA	186	60
*HIRF*^b^	Forward: TTACCAGTGCCTCTCCTCCAT Reverse: GCGTGACAAACCCCAGTTATCT	127	60
*CFP*	Forward: TTCCCGCCCACCATTACC Reverse: GCCGTTTCTCCTCCACCATC	121	60
*HP*	Forward: ACTCCACGGTAGACATCGG Reverse: GTTGAGGTTGGCGTTTCG	149	63
*S100A8*	Forward: GCGTAGATGGCGTGGTAA Reverse: GCCCTGCATGTGCTTTGT	155	60
*THY1*	Forward: CGGCTATTCGTCCTCTTTGTT Reverse: CCGACCCTGGCTTCCCTT	102	60
*CCL5*	Forward: CCTGAGACAGCCCGTGGAT Reverse: GGTGTGGTGTCCGAGGCAT	141	60
*ERAF*	Forward: GCCCTTCTTCCAACCAATCA Reverse: CCCCCACCATCTTCTTCTTGTAG	172	60
*RPL32*^c^	Forward: CGGAAGTTTCTGGTACACAATGTAA Reverse: TGGAAGAGACGTTGTGAGCAA	94	60–63

^a^The annealing temperature represents the optimal temperature during quantitative PCR. ^b^HPS infection related factor; No significant similarity was found after BLASTX searches and we named this gene ourselves. ^c^RNA levels of RPL32 was assayed for normalization during quantitative PCR.

### Induction of other genes related to defense/immunity after HPS infection

In total, five genes that are associated with regulation of transcription were differentially expressed in HPS-challenged porcine spleen (Table 2). Two members of the CEBP family, *CEBPD *(CCAAT/enhancer binding protein delta, 4.7-fold) and *CEBPB *(CCAAT/enhancer binding protein beta, 3.1-fold), which are crucial in the regulation of genes involved in immune and inflammatory responses, were up-regulated. In addition, up-regulation of *ZBTB16 *(zinc finger and BTB domain-containing protein 16, 5.6-fold) and *SLC39A14 *(zinc transporter solute carrier family 39 member 14, 4.5-fold) was also observed, suggesting that zinc finger transcription factors may play an important role during the pathogenesis of HPS infection.

We also identified many genes that are related to oxidative stress, homeostasis, and wounding (Table 2). *SOD2 *(superoxide dismutase 2, 3.8-fold up-regulation) provides vital protection against reactive oxygen species (ROS), thus protecting tissues from damage in a broad range of disease states. Additionally, this enzyme can efficiently catalyze the formation of bactericidal H_2_O_2 _from the superoxide radical anion. The GO terms "cell ion homeostasis", "iron ion homeostasis", "metal ion homeostasis", and "di-, tri-valent inorganic cation homeostasis", etc. (Figure 4), were shared by the same genes. In addition to *SAA1*, *LTF*, and *CP*, which have been grouped with the acute-phase response proteins, *TCN1 *(transcobalamin I), which is a major constituent of secondary granules in neutrophils and facilitates the transportation of cobalamin into cells, was also up-regulated dramatically, at 17.7-fold.

*HLA-B *(major histocompatibility complex (MHC); class I; B) and *HLA-DRB1 *(MHC; class II; DR beta 1) are two genes that are involved in antigen presentation. Interestingly, both of the MHC genes, which connect the innate immune system with the adaptive immune system, were down-regulated, at ≈3.3-fold and 2-fold respectively. The DE genes encoding transcription factors, oxidative stress, homeostasis, and wounding, etc., indicate that many immune-related signal pathways were activated and that immune surveillance was occurring in the infected porcine spleen.

### Induction of genes related to cell development

Analysis of GO, together with manual annotation, showed that 21 genes that were classified into the subset related to "development" were induced after infection (Figure 4 and Table 2). The most highly induced genes included the actin-binding phosphoprotein *CLF1 *(cofilin 1),*P8 *(p8 protein), and *NAV1 *(neuron navigator 1) (19.4-, 5.4-, and 3.4-fold, respectively). In contrast, many genes in this group showed dramatic down-regulation, including *ERAF *(erythroid associated factor, 100-fold), *CRABP1 *(cellular retinoic acid binding protein 1, 10-fold), *ACTC1 *(actin; alpha; cardiac muscle, 3.3-fold) and many others. Interestingly, *ERAF *has been reported to be a novel erythroid-specific marker of transmissible spongiform encephalopathies (TSE). As in our study, it has been found to be decreased dramatically in spleen during the terminal stages of TSE [20]. The change in expression of *ERAF *was further confirmed by quantitative PCR (QPCR) analyses (see next section). The DE genes related to cell development are likely to be connected to the series of histomorphologic changes that occur during the course of disease, or to the regulation of the immune system during stress.

### Validation of microarray data by QPCR

**Figure 5:**
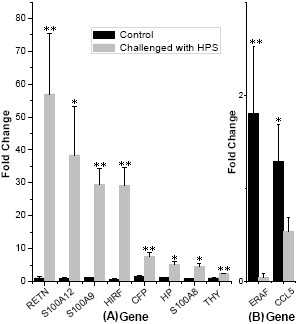
**Validation of the microarray data by QPCR analyses**. *RETN*, *S100A12*, *S100A9*, *HIRF*, *CFP*, *HP*, *S100A8*, and *THY *(A) were up regulated in HPS infected piglets (light gray bars) compared with normal controls (black bars), *CCL5 *and *ERAF *(B) were down regulated. Fold change is calculated based on the mean intensity value from 3 donors within each group by using the comparative Ct method. Significant levels were analyzed by T-test. **, *p *≤ 0.01; *, *p *≤ 0.05.

In order to validate the differential expression of various genes identified using the microarray, eight up-regulated genes, with increases ranging from 1.6-fold to 111.4-fold, and two down-regulated genes, with decreases ranging from 2.5-fold to 100-fold, were selected for QPCR analyses. *THY1 *(thy-1 cell surface antigen) and *CFP *(complement factor properdin), which showed changes of less than 2-fold in the microarray results, and *HIRF *(HPS infection related factor), which was not annotated in the analysis of GO, were also selected for QPCR confirmation (Table 3). Genes with no statistical significance were not involved in the QPCR analyses. All the selected genes showed significant (*p *< 0.05) differential expression in the QPCR results (Figure 5). The FCs measured by QPCR were either much greater or much smaller than those obtained using the microarray (Table 4). This was probably due to the greater accuracy of quantitation provided by QPCR in comparison to microarrays, the differences in the dynamic range of the two techniques, and the lack of specificity in the primers designed to discriminate gene family members at the level of primary screening by DNA arrays. However, the trends were coincident between the results of the two techniques, which indicates the reliability of our microarray analyses.

**Table 4:** A validation of microarray data by QPCR

Gene	GenBank ID	Microarray FC	QPCR FC	*p*-value
*RETN*	AY488504	+^a ^25.9	+55.3	9.E-03
*S100A12*	AK230982	+16.6	+48.9	2.E-02
*S100A9*	CB4772207	+21.8	+26.7	8.E-03
*HIRF*	BI402280	+111.4	+41.0	1.E-03
*CFP*	AK232839	+1.6	+5.6	9.E-03
*HP*	NM214000	+10.0	+4.8	5.E-02
*S100A8*	CB474992	+8.4	+5.9	3.E-02
*THY1*	AK233381	+1.6	+2.2	8.E-03
*CCL5*	DQ372066	-^b ^2.5	-2.5	2.E-02
*ERAF*	CF788119	-100.0	-39.9	1.E-02

^a^Represents up regulation. ^b^Represents down regulation.

### Expression analyses of *S100A8*, *S100A9*, *S100A12*, and *HP *in PK-15 cells stimulated with LPS and Poly(I:C)

*HP *is a plasma a2-glycoprotein that can bind free hemoglobin and prevent oxidative damage [21]. It is considered to be an important marker of inflammation [22]. S100A8, S100A9, and S100A12 belong to the S100 family, which are also known as myeloid-related proteins (MRPs). These three EF-hand homolog S100 proteins play a pivotal role in the innate immune system and have been considered for use as clinical laboratory markers of inflammation [23]. Interestingly, S100A9 and S100A12 have been reported to be up-regulated in peripheral blood mononuclear cells (PBMCs) during severe acute respiratory syndrome (SARS) [24]. These three MRPs can also induce transcriptional activity and viral replication of HIV-1 in infected CD4^+ ^T lymphocytes [25]. These observations drove us to investigate further their expression patterns under general conditions that mimic bacterial and viral infections.

**Figure 6:**
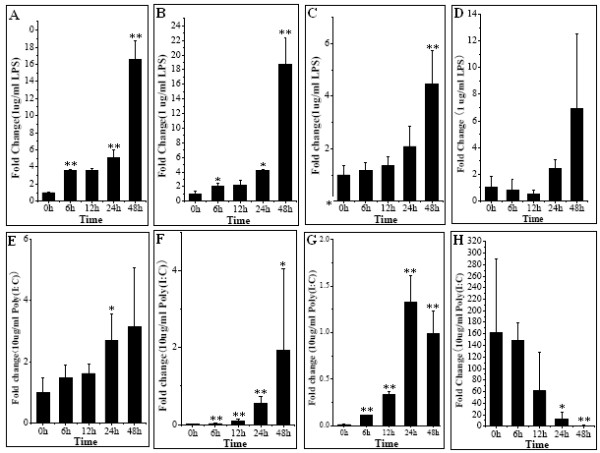
**Kinetic immune stimuli analyses challenged by LPS(A-D) and Poly(I:C)(E-F) in PK-15 cells**. S100A8(A, E), S100A9(B, F), S100A12(C, G), and HP(D, H). PK-15 cells were treated with 1 μg/mL LPS and 10 μg/mL Poly(I:C) respectively. QPCR was performed using primers described in Table 3. Results are from the calculated average ± SD of three different cell samples in the same treatment. The significance of differences for the expression compared to the untreated control was calculated using two-directional paired Student's T-test. **, *p *≤ 0.01; *, *p *≤ 0.05.

Our results demonstrated that these three MRPs had similar expression tendencies following treatment with lipopolysaccharide (LPS) and polyinosinic acid-polycytidylic acid (Poly(I:C)) in pig kidney-15 (PK-15) cells (Figure 6A–C, and 6E–G). However, there were some differences in expression between the two treatments. Firstly, *S100A8 *was up-regulated at an earlier time following LPS challenge, and time dependence was observed (Figure 6A). In contrast, it was up-regulated at a later time in PK-15 cells stimulated by Poly(I:C) (Figure 6E). The expression pattern of *S100A12 *was reversed in comparison with that of *S100A8 *(Figure 6C and 6G). Nevertheless, it seems that both LPS and Poly(I:C) induced a more significant time-dependent up-regulation in the expression of *S100A9 *than that of *S100A8 *(Figure 6B and 6F). Secondly, the transcription levels of *HP *tended to show an increase following LPS treatment (Figure 6D), which is appropriate for an important acute-phase protein. However, we found a marked decreased level of transcription of *HP *mRNA following challenge with Poly(I:C) (Figure 6H). Thirdly, the FCs differed between the treatments with LPS and Poly(I:C) at the same time point. This is probably attributable to the dose-response relationship of each treatment. Our observation, along with the results presented in many other reports, increases our understanding of the importance of MRPs and HP during the innate immune response.

### Identification of DE genes in known pig QTL regions associated with health traits

**Figure 7:**
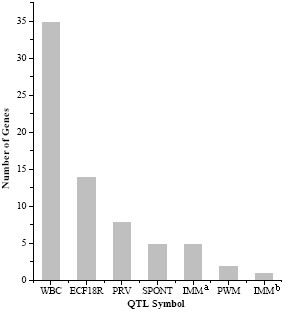
**Genes that distributed in the known pig QTLs of Health Traits**. WBC, White Blood Cell Counts; ECF18R, Small Intestinal Escherichia Coli F18 Receptor; PRV, Resistance/Susceptibility To Pseudorabies; SPONT, Spontenous Cell Proliferation; IMM^a^, Stress-Induced Alterations In Mitogen Induced IL-2 Activity; PWM, PWM Induced Cell Proliferation; IMM^b^, Stress-Induced Alteration In Number Of Neutrophils; These seven QTLs belong to two big categories of the health traits: WBC, SPONT, IMM^a^, PWM, IMM^b ^are grouped into the category of "Pathogen Immune Capacity"; ECF18R and PRV are grouped into the category of "Disease Resistance".

Quantitative genetic analysis suggests that many traits related to disease resistance are determined by quantitative trait loci (QTLs). The ultimate goal of QTL studies is the identification of the actual gene(s) that are responsible for the phenotypic variation observed in a particular trait [26]. The recently established Animal Quantitative Trait Loci (QTL) database (AnimalQTLdb) has offered new opportunities to search for positional candidate genes or markers that underlie the QTL region of a particular trait [27]. Our search found that 70 DE genes (*p *< 0.05) were distributed in seven different known QTL regions related to pig health traits (Figure 7 and Additional file 4). It is noteworthy that 14 annotated genes that showed changes of more than 2-fold were distributed in five different QTL regions (see Additional file 4). Interestingly, the inflammatory marker *HP *was found to be linked to PRV, which is a QTL region associated with resistance/susceptibility to pseudorabies. The transcriptional factor *CEBPD *was found to be located in the QTL region of the small intestinal *Escherichia coli *F18 receptor (ECF18R). In addition, serpin peptidase inhibitor, clade E, member 2 and interleukin 10 receptor beta were found to be linked to white blood cell counts (WBC) and spontaneous cell proliferation (SPONT), respectively. This work may provide important clues or evidence that allow us to explain gene functions, and further, to explore the pathogenesis of infection associated with innate immune response.

## Discussion

HPS is an important pathogen of swine that has effects both on the pork industry and on animal welfare. As with all bacteria, the use of antibiotics to treat this pathogen, and the subsequent development of antibiotic resistance, is becoming another potential threat to food safety and human health. New trends in the diagnosis, epidemiology and control of HPS have been well reviewed [6]. Although great efforts have been made by many researchers, the molecular basis of this infection is largely unknown. Advances in genomics have allowed researchers of pig genetics to examine changes in gene expression in organisms that are subjected to infections with a variety of pathogens through high throughput microarray technology [28]. Microarray studies have provided sets of genes that could serve as natural targets for further functional characterization. In the present study, the global transcriptional response of porcine spleen to HPS has been reported for the first time. In particular, 92 unique genes with known functions showed specific responses to HPS. The other DE genes on the Affymetrix GeneChip Porcine Genome Arrays were not annotated because of the limited availability of full-length porcine cDNA and the unknown functional annotations of human and murine genes.

The spleen is the largest lymphatic organ in the pig. Many immune cells, including B cells, T cells, natural killer (NK) cells, dendritic cells, macrophages, etc., exist in the spleen. The immune system of the spleen has been proven to play a fundamental role in protecting the body from invading pathogens and in the detection of senescent, mechanically damaged, displaced and aberrant cells that could lead to the formation of tumors. It is accepted that the spleen has the dominant role in the simultaneous dual reaction to bacterial antigens. Both an immediate innate reaction and an adaptive immune response, which involves interaction between cells that recognize a particular antigen, can be invoked during microbial penetration of tissues [29]. Recently, much progress has been made towards discovering the common mechanisms that underlie microbial pathogenesis [30]. There is no doubt that many cascades that are accepted to be involved in innate or adaptive immunity against the Gram-negative bacterium HPS also take place in the spleen.

The severe inflammation presented during the pathogenesis of HPS infection may explain why the inflammasomes are important. *RETN *was the most highly up-regulated gene among several genes that are involved in the inflammatory response (Table 2), and the high expression levels were further confirmed by QPCR (Figure 5). It is a member of the adipokines, which have been found to be biologically active mediators present in the adipocytes of white adipose tissue [18]. Nevertheless, this cytokine has also been found to be widely expressed in human PBMC, macrophages, and bone marrow cells [31,32]. In the pig, *RETN *has been cloned [33] and mapped to chromosome 2 [34]. Kougias et al. reported that porcine RETN causes endothelial dysfunction of coronary arteries [35], but its other functions are not clear. In humans, great efforts have been made in the last few years towards identifying the role of RETN in the inflammatory response. It can be induced in PBMCs by LPS, IL-1, IL-6, and TNFα, probably through activation of NF-κB cells. Stimulation with RETN leads to dramatic elevations of IL-6, TNFα, and IL-1β [36,37], which suggests that a positive feedback mechanism exists between RETN and some key cytokines. In addition, many other cytokines, e.g., IL-8 and IL-12, adhesion molecules, e.g., VCAM1 and ICAM1, and TLR2, etc., can be induced by this adipokine in many different cell types [38]. It is worth underlining that resistin has been suggested to be involved in vascular inflammation and dysfunction [39], and its involvement in rheumatoid arthritis has been generally accepted [40]. Therefore, RETN is a proinflammatory cytokine derived from systemic immunity that targets both innate immunity and systemic diseases.

Using a combination of the microarray and QPCR, we also identified another set of inflammasomes: S100A8, S100A9, and S100A12. Wang et al. reported that these genes were up-regulated in porcine mesenteric lymph notes in response to *Salmonella enterica *serovar Typhimurium [28]. These intracellular molecules that bind calcium are thought to become pro-inflammatory danger signals after release into the extracellular compartment under conditions of cell damage, infection or inflammation [41]. It has been reported that S100A8/S100A9 recruits leucocytes by increasing the binding capacity of the integrin receptor CD11b-CD18 on leucocytes to ICAM-1 in the endothelium [42]. Murine S100A8 can induce phagocytes to express TNF, IL-1β, and IL-12 in a TLR4-dependent manner [43]. In addition, IL-1 can induce the secretion of S100A8/S100A9 by human monocytes [44,45]. S100A12 has been proven to be a member of a novel inflammatory signaling pathway that involves the receptor for advanced glycation end products (RAGE) [46]. Furthermore, adhesion molecules as well as pro-inflammatory cytokines, e.g., VCAM-1, ICAM-1, IL-8, and MCP-1, can be induced by S100A12 in endothelial cells in an NF-κB-dependent manner [47]. The platelet aggregation protein thrombospondin 1, which was up-regulated 2.2-fold in our microarray data, indicates that the thrombogenic response induced by S100A8/A9 in endothelium may have been activated following HPS infection. Of note, the emphasis of research into the three inflammasomes has been focused on determining the principal factors responsible for inflammatory arthritis and systemic juvenile idiopathic arthritis [41], which provides another clue for investigating their effects on porcine arthritis caused by HPS. Interestingly, LPS and Poly(I:C) induced large increases in the expression of these three molecules in PK-15 cells at the mRNA level (Figure 6). This response was sustained, which suggests that these proteins may have wide involvement in host defense. Together with other inflammasomes (Table 2), they may have extensive roles in the clearance of HPS and the pathogenesis of systematic inflammation during HPS infection. Further studies are required to elucidate the details of the mechanisms involved.

Acute-phase proteins (APP) and complement constitute another notable subsystem/module used by the porcine innate immune system. These genes include *SAA1*, *LTF*, *AGT*,*HP*, *CP*, and *C3*, which were found during HPS infection at levels far exceeding those found in control individuals. Interestingly, the last five were reported to be differentially expressed in fish following bacterial challenge [48], indicating the likely evolutionary conservation of function of these APPs between different species.*SAA1 *is a member of the human SAA family. This APP has been ascribed numerous immune effects that are involved in chemotactic activity and the production of cytokines. Recent data have demonstrated that SAA has the ability to induce antiapoptotic effects [49,50], in keeping with its proinflammatory cytokine properties. Interestingly, Lee et al. reported that SAA stimulates the production of the proinflammatory and anti-inflammatory cytokines TNFα and IL-10 via activation of NF-κB downstream of FPRL1 (formyl peptide receptor-like 1) activation [51]. Additionally, it has been reported that SAA functions as an opsonin that facilitates the phagocytosis of Gram-negative bacteria [52]. Apart from the S100 proteins mentioned above, SAA proteins have also been detected in patients with various forms of inflammatory arthritis [53]. Given that the RAGE pathway has been suggested to be important in systemic amyloidosis, the potential S100A12-RAGE-SAA1 cascade may exist in pigs with arthritis caused by HPS infection.

Iron is an essential nutrient for most microbial pathogens. Our results indicate that the innate immune system of the pig may limit the growth of HPS by using *LTF *and *HP *during HPS infection. Besides its iron-binding properties, LTF has also been shown to have antimicrobial activities that are independent of iron status; it prevents the adhesion and internalization of bacteria and viruses to tissue cells [54] and enhances the intracellular bactericidal activity of neutrophils [55]. Of note, the synergic antistaphylococcal properties of LTF and LYZ (Table 2) have been demonstrated. In particular, they have the ability to kill Gram-negative bacteria, including *Vibrio cholerae*, *S*. Typhimurium, and *E. coli *[56-58]. Although there is no direct evidence that they can kill HPS, we presume that they can be bactericidal for HPS during infection because of the similar outer membranes of all Gram-negative bacteria. The protein HP, together with CD163, plays an important role in the modulation of both inflammatory and immune responses. Given that both HP and hemoglobin are degraded in the lysosome after endocytosis of the HP-haemoglobin-CD163 complex by macrophages [21], LYZ may be a key "partner" in this process as well. In addition, treatment with LPS and Poly(I:C) in PK-15 cells showed different expression patterns of *HP *(Figure 6). Elevation in *HP *levels, e.g., after HPS infection, may generate a feedback effect that reduces the severity of the acute-phase reaction [59]. However, we found it difficult to explain why *HP *was down-regulated in PK-15 cells treated with Ploy(I:C). Is the reduction in *HP *mRNA levels related to an unknown feedback mechanism that underlies the degradation of HP in the lysosome? Or is it possible that the sensitivity of PK-15 cells to many inflammatory mediators (e.g., IL-1, IL-6, TNF) has been modified by Poly(I:C)? It should be emphasized that *HP *is becoming an important candidate gene in both human and porcine diseases and our bioinformatics analyses of pig QTLs associated with health traits (Figure 7) also suggested that *HP *is located in the pig QTL for PRV (see Additional file 4). Activation of the complement system also plays a critical role in the eradication of pathogens. Elevation of C3, the central component of the complement system, in our results may indicate its involvement in the acute-phase and inflammatory processes that occur during HPS infection. Unfortunately, this is the only guesswork based on the published information [60], and it is yet unclear whether the activities of the APPs detected by our assay are related to activation of complement.

One of the highlights of our study is that several genes responsible for the differentiation of epithelial cells and keratinocytes were identified; of these, *TGM1*, *MS4A8B*, and *CSTA *were highly induced. *TGM1 *is a member of the transglutaminase family, which can stabilize biological structures by catalyzing the formation of ε-(γ-glutamyl)-lysine crosslinks in proteins [61]. Given that it is normally expressed in many types of epithelial cell, we hypothesize that it may prevent the host from HPS infection. Of note, TGM1 is dramatically up-regulated in porcine lung infected with *Salmonella *Choleraesuis [62], which suggests that it may have an important role in the porcine immune response to Gram-negative bacteria. We herein first report the detection of *MS4A8B*, a member of the MS4A family, which is structurally related to CD20, FceRIb, and HTm4. *MS4A8B *can be expressed by spleen, bone marrow, peripheral lymph node, and many other tissues but probably only by B cell lines [63]. It has been identified recently as a novel gene in present in bronchial epithelium, and is presumably linked to respiratory function [64]. *CSTA *belongs to the cystatin family, which are natural inhibitors of cysteine cathepsins and have been recently proposed to induce production of TNF, IL-10, and nitric oxide [65]. Previous studies suggest that *CSTA *plays a critical role in the proliferation and differentiation of keratinocytes, and in regulating antigen presentation and apoptosis [66-68]. Importantly, down-regulation of *CSTA *has been detected in a wide range of epithelial tumors [69], which further indicates its important role in the differentiation and development of epithelial cells.

So far, little has been reported of the two adhesion molecules *CLDN3 *and *CSPG2 *in pigs. CLDN3 plays a major role in the formation of tight junctions (TJs), and it has been demonstrated that CLDN3 is a receptor of the *Clostridium perfringens *enterotoxin (CPE) [70]. The CPE, upon binding to CLDN3, causes cytolysis through its effects on membrane permeability. Recent data suggest that *CSPG2*, also named *VCAN *(versican), modulates cell adhesion, proliferation, and migration, and plays a central role in the development and pathogenesis of several diseases, including cardiovascular diseases [71]. CSPG2 also binds to the cell surface protein CD44 [72], another adhesion molecule, which was up-regulated 2.5-fold in our assay. Interestingly, the novel ligand of CD44, LGALS8/galectin-8, was also elevated significantly. Galectin-8, along with CD44, has been reported to be of great importance in the regulation of autoimmune inflammation by apoptosis [73]. Additionally, data suggest that galectin-8 can modulate the function of neutrophils [74] and can induce apoptosis in the CD4^high ^CD8^high ^subpopulations of thymocytes [75]. Taken together, the up-regulation of several adhesion molecules during HPS infection may have a wide spectrum of immune functions, and synergies may further reinforce the functions under some conditions.

We also observed that several transcription factors showed increased expression following HPS challenge (Table 2). *ZBTB16 *(also known as promyelocytic leukemia zinc finger protein, *PLZF*) encodes a zinc finger transcription factor that regulates embryonic development of the skeletal and central nervous systems, and spermatogenesis; it has also been associated with apoptosis and tumor repression [76]. Recent studies suggest that this transcription factor participates in the novel (P)RR-PLZF-PI3K-p85α signal transduction cascade, which is related functionally to the renin-angiotensin system [77]. Interestingly, the inflammasome RETN is also linked to the PI3K-p85α cascade [78], which indicates that crosstalk may exist between these key molecules. Although we did not find IL-6 on the array, elevation of *SLC39A14 *indicates that *IL-6 *may has been up-regulated during HPS infection. This notion is supported by the discovery that IL-6 induces an increase of SLC39A14 in the liver and contributes to the acute-phase response [79]. CEBPD and CEBPB may act as key transcription factors that are involved in the various modules mentioned above. They belong to the C/EBP family, which regulates a number of genes that are involved in innate immunity, cell proliferation, adipogenesis, and the inflammatory and acute-phase responses [80]. Four genes, *RETN*, *SAA1*, *SOD2*, and *P8*, which were up-regulated following HPS infection, are known to be regulated by C/EBPs [37,81-83]. In particular, *SAA1 *and *P8*, whose promotor regions contain the sites that bind to the transcription factor CEBPB. Our QTL analyses showed that *CEBPD *may also be linked to ECF18R (see Additional file 4), a crucial protein that counteracts *E. coli *infection in young piglets, which indicates its important role in innate immunity.

Last, but not least, two MHC genes, *HLA-B *and *HLA-DRB1*, which are named *SLA-2 *(swine leukocyte antigen-2) and *SLA-DRB1 *in the pig, respectively, are especially significant. Many genes from pigs that encode MHC class I and class II molecules have been extensively characterized [84,85], but little is known about their expression patterns following infections with pathogenic organisms. The down-regulation of both *HLA-B *and *HLA-DRB1 *indicates that weakened processing and presentation of antigens were likely to occur in pigs 7 days after HPS infection, as part of the cell-mediated immune response. Hence, responses mediated by both MHC class I with CD8^+ ^cytotoxic T lymphocyte (CTL) and MHC class II with CD4^+ ^T-helper (T_H_) cells would be expected of HPS-infected cells in the spleen. Especially important in this regard are responses of CD4^+^αβ T cells, because of their predominant role in bacterial and parasitic infections in pigs [86]. The down-regulation of the two MHC genes may also indicate a potential immune evasion of HPS. We suspect that the evasion may be reinforced by suppression of T cell responses by HPS. This hypothesis could be further confirmed by the down-regulation of the T-cell differentiation protein mal (*MAL*) and the intracellular signal transduction protein PDZ domain containing 2 (*PDZD2*) (by 5-fold and 3.3-fold respectively, Table 2). Both of these proteins are localized to the endoplasmic reticulum, which is an important location for processing and presentation of peptides during the development of acquired immunity. Nevertheless, to unmask the mechanism of immune evasion of HPS may be far more complicated than we thought. Further studies based on genetics and cell biology are clearly needed in pigs to understand the role of the MHC and T cell-mediated response to HPS infection, and to unravel the molecular basis for disease resistance to Glässer's disease in swine.

## Conclusion

We have identified the global changes in gene expression in porcine spleen that follow challenge with HPS. Although the work was limited to three animals in each group and to a single time point, the present microarray analysis provides new information that increases our understanding of the infection with the Gram-negative bacterium HPS. Our data show that a series of genes that are involved in immune responses are activated following HPS invasion, particularly genes of the inflammatory and acute-phase responses. This finding could contribute to explaining the complicated mechanism of systematic inflammation that underlies the pathogenesis of the disease. In addition, the down-regulation of genes that are associated with antigen processing and presentation could be responsible for immune evasion of HPS. More generally, the microarray data are likely to be helpful in uncovering potential crosstalks regarding mammalian immune response mechanisms. In addition, a number of unknown genes also showed significant differential expression patterns following HPS challenge, which indicates that many novel porcine genes remain to be characterized. Taken together, we our results add potential insights into therapies for HPS and the molecular basis of disease resistance in the pig as well as provide fundamental information regarding mammalian immune response mechanisms.

## Methods

### Animals and tissue collection

All animal tissue collection procedures were performed according to protocols approved by The Hubei Province, PR China for Biological Studies Animal Care and Use Committee. 10 piglets which were obtained from a commercial herd free of Glässer's disease were weaned at 10 days, shipped to the Animal Disease Center of Huazhong Agricultural University, and raised in isolation facilities. At 30 days of age, 5 pigs were randomly allocated to the noninfected group and 5 to the infected group. Each piglet of the infected group was intratracheally challenged with HPS strain 0165 (serotype 5) at a dose of 5 × 10^8 ^colony-forming units (CFU). Each piglet of the noninfected group was treated similarly with an identical volume of PBS as control. All piglets were determined to be HPS-free by serum indirect haemagglutination (IHA) test before artificial bacterial challenges [87]. Clinical signs and lesions of Glässer's disease were apparent in the challenged group after 7 days post infection (dpi). All pigs were slaughtered at 7 dpi. Bacteria isolation and HPS specific PCR by 16S rRNA [88] were performed after the pigs were killed at 7 dpi. The infected group showed positive, and the control group was negative. 10^5^–10^7 ^CFU bacteria per gram of tissue were re-isolated from brains and lungs of the infected group, but not from those of control group. Spleens from both groups were snap frozen in liquid nitrogen after collection and stored at -80°C until RNA extraction. TRIzol (Invitrogen) and RNeasy Mini Kit (QIAGEN) were used for RNA extractions and purifications respectively followed by manufacturer's instructions. RNA integrity and concentration were evaluated by Agilent 2100 Bioanalyzer.

### Microarray hybridizations and data analyses

The RNA labelling and hybridization were conducted by a commercial Affymetrix array service (GeneTech Biotechnology Limited Company, Shang Hai, China). Briefly, a total of 5 μg RNA was converted to double-stranded cDNA using the one-cycle cDNA Synthesis Kit (Affymetrix, Inc., Santa Clara, CA) and an oligo-dT primer containing the T7 RNA polymerase promoter.*In vitro *transcription (IVT) of cRNA from cDNA was conducted using the MEGAscript^® ^T7 Kit (Ambion, Inc.). The cDNA and cRNA were purified using the Sample Cleanup Module (Affymetrix). The GeneChip IVT Labeling Kit (Affymetrix) was used for Synthesis of Biotin-Labeled cRNA. cRNA quality and concentration was checked by UV spectrophotometer analyses and 2 μg cRNA was then checked by formaldehyde denaturing gel electrophoresis in 1.2% agarose gel characterized by dispersed straps (28S and 18S) without any obvious smearing patterns from degradation. Subsequently, labeled cRNA was fractionated and hybridized with the GeneChip Porcine Genome Array according to the standard procedures provided by the manufacturer. Chips were washed and stained with a GeneChip Fluidics Station 450 (Affymetrix) using the standard fluidics protocol. The probe arrays were scanned using the Affymetrix^® ^GeneChip^® ^Scanner 3000. Six microarrays were used in the experiment, corresponding to the RNAs from spleen tissues of three HPS infected piglets and three controls.

There are 11 paired perfect match (PM) and mismatch (MM) 25-mer probes in the Affymetrix GeneChip porcine genome array for each probeset representing a gene. The signals from these probe pairs are used to determine whether a given gene is expressed and to measure the gene expression level. The probe-pair (PM-MM) data were used to detect the expression level of genes on the array (present call, marginal call, and absent call) by MAS 5.0 (Wilcoxon signed rank test). Raw data from .CEL files were converted to gene signal files by MAS 5.0 (Ver.2.3.1). Natural logarithms were then taken on quantile normalization (GeneSpring 7.31). Identification of DE genes was conducted using SAS (Ver.8.1, T-test). The p value cutoff for DE genes was set at 0.05. The data discussed in this publication have been deposited in NCBI's Gene Expression Omnibus and are accessible through GEO Series accession number GSE11787 . DE genes were performed for hierarchical cluster (Ver.2.11) and TreeView (Ver.1.60) analyses [89]. Gene ontology (GO) analyses was conducted at the DAVID [90]. Genes with significant similarities to transcripts in *nr *database based on BLASTX searches were selected for GO analyses. Annotation summary results were obtained by inputting the gene list of interest by selecting GENBAMK_ACCESSION as identifier.

### In vitro LPS and Poly (I:C) stimulation of PK-15 cells

PK-15 has been proved to be especially useful for study of infectious disease processes in swine [91]. In this study, ten groups (with three repeats in each group, ~1 × 10^5 ^cells/sample) of PK-15 cells from the same cell culture flask were grown in culture medium (DMEM) supplemented with 10% heat-inactivated fetal bovine serum at 37°C with 5% CO_2_. Adherent PK-15 cells were obtained by washing off nonadherent cells with warm culture medium and PBS twice, respectively. Adherent cells were further cultured in DMEM (control samples) or treated with 1 μg/mL LPS (Sigma, *E. coli *0127:B8) or 10 μg/mL Poly(I:C) (Sigma) respectively (stimulated samples) for 0 h, 6 h, 12 h, 24 h, 48 h. Cells were harvested and total RNA was extracted as described above.

### QPCR analyses

Both spleen RNA prepared for microarray analyses and RNA from LPS and Poly(I:C) stimulated PK-15 cells were used for QPCR analyses. Total RNA were treated with DnaseI (Tubo kit, Ambion) and reverse transcribed by the M-MLV Reverse Transcriptase (Promega) according to the manufacturer's instructions. Amplification of the ribosomal protein L32 (RPL32) gene was used as the internal control. QPCR was performed on the iQ™5 Real Time PCR Detection System (Bio-Rad) using SYBR^® ^Green Realtime PCR Master Mix (TOYOBO CO., LTD, Japan) as the readout. PCR conditions were: 95°C, 3 min ; 95°C, 30 s, 59.8–63°C as appropriate, 30 s, and 72°C, 15 s for 40 cycles. Melt curves were obtained by increasing the temperature from 55°C to 95°C at 0.5°C/s for 10 s, then cooling at 25°C for 30s. The primers were designed by the program Primer 5.0. The primer sequence, melting temperature and product sizes are shown in Table 3. The correct fragment sizes of the PCR products were confirmed using agarose gel electrophoresis (1.5%). Each primer set amplified a single product as indicated by a single peak present for each gene during melting curve analyses. The relative quantitative gene expression level was evaluated using the comparative Ct method [92]. The Δ*Ct *values were calculated by subtracting the RPL32 *Ct *value for each sample from the target *Ct *value of that sample. The duplicates for each sample were averaged, Δ*Ct *values were calculated, and pairwise *t *tests were conducted on these averages to identify genes differing in expression between control and treatment. In all QPCR analysis, *p *< 0.05 was considered significant.

### Mapping of the DE genes to pig QTLs regions of health traits

All health traits could be browsed by trait classes in Pig Quantitative Trait Loci database (PigQTLdb). Mapping details could be obtained by clicking on the QTL name on the corresponding pig chromosome. The pig Affymetrix elements corresponding to health trait QTL regions were downloaded to an excel file. DE genes that located in the corresponding QTL region could be obtained by matching the ID of DE genes (all the 931 transcripts that were identified at the *p *< 0.05 level) to all the genes in the QTL regions using the software JMP (Ver.5.1.2).

## Abbreviations

HPS: *Haemophilus parasuis*; DE: differentially expressed; FC: fold change; GO: Gene Ontology; MHC: major histocompatibility complex; QPCR: quantitative PCR; LPS: lipopolysaccharide; Poly(I:C): polyinosinic acid-polycytidylic acid; PK-15: pig kidney-15; QTL: quantitative trait loci.

## Authors' contributions

HC and CL carried out all works in the lab and drafted the manuscript. MF and MZ made substantial contributions to bioinformatics and statistical analysis. XL and RZh participated in the animal challenge experiment and immunoassays. KL participated in the experiment design and coordination. SZ conceived the study, and participated in its coordination and helped to draft the manuscript. All authors read and approved the final manuscript.

## Supplementary Material

Additional file 1 **Spleen transcriptome analyses following Haemophilus Parasuis infection using the Affymetrix Porcine Genechip.** Data of each probe is from three piglets of the infection group (Infection1, Infection2, Infection3) and the control group (Control 1, Control 2, Control 3). Data of the control group is displayed in bold and names of probes are listed on the left of each group. Raw data from .CEL files were converted to gene signal files by MAS 5.0 (Ver.2.3.1), natural logarithms were then taken on quantile normalization (GeneSpring 7.31, "P", present; "A", absent; "M", marginal; "Count(P)", the number of P flag; select while Count(P)≥2 in two groups. Totally, 13,833 and 13,857 probesets were detected expression in the infection group and the control group respectively. Click here for file

Additional file 2 **931 transcripts that are differentially expressed in porcine spleen following Haemophilus Parasuis infection.** A total of 682 (row 8–689) transcripts have been annotated based on BLASTX searches. "FC", Fold change, gene expression level following infection compared to the control. "≥2" represents up regulation, "<1" represents down regulation. "p-value", significance level of differential expression for a particular gene. "Gene description", top informative BLASTX hit. Click here for file

Additional file 3 **Unique genes with BLSATX identity but no annotation (row 7–72) and unique genes with no BLASTX identity (row 73–161) (p < 0.05, FC≥2).** "FC", Fold change, gene expression level following infection compared to the control. "≥2" represents up regulation, "<1" represents down regulation. "p-value", significance level of differential expression for a particular gene. "Gene description", top informative BLASTX hit. Click here for file

Additional file 4 **Genes that distributed in the known pig QTL regions of Health Traits. **All the genes listed here are from 931 transcripts that are significantly up/down-regulated following Haemophilus Parasuis infection. GenBank IDs represent annotated genes, and UniGenes represent genes with no annotation. 14 annotated genes with more than 2-fold changes are listed in bold. Click here for file
